# Effects of Data Augmentation on the Nine-Axis IMU-Based Orientation Estimation Accuracy of a Recurrent Neural Network

**DOI:** 10.3390/s23177458

**Published:** 2023-08-28

**Authors:** Ji Seok Choi, Jung Keun Lee

**Affiliations:** Inertial Motion Capture Lab, School of ICT, Robotics & Mechanical Engineering, Hankyong National University, Anseong 17579, Republic of Korea; 2021585203@hknu.ac.kr

**Keywords:** data augmentation, orientation estimation, inertial measurement unit, deep learning, recurrent neural network

## Abstract

The nine-axis inertial and measurement unit (IMU)-based three-dimensional (3D) orientation estimation is a fundamental part of inertial motion capture. Recently, owing to the successful utilization of deep learning in various applications, orientation estimation neural networks (NNs) trained on large datasets, including nine-axis IMU signals and reference orientation data, have been developed. During the training process, the limited amount of training data is a critical issue in the development of powerful networks. Data augmentation, which increases the amount of training data, is a key approach for addressing the data shortage problem and thus for improving the estimation performance. However, to the best of our knowledge, no studies have been conducted to analyze the effects of data augmentation techniques on estimation performance in orientation estimation networks using IMU sensors. This paper selects three data augmentation techniques for IMU-based orientation estimation NNs, i.e., augmentation by virtual rotation, bias addition, and noise addition (which are hereafter referred to as *rotation*, *bias*, and *noise*, respectively). Then, this paper analyzes the effects of these augmentation techniques on estimation accuracy in recurrent neural networks, for a total of seven combinations (i.e., rotation only, bias only, noise only, rotation and bias, rotation and noise, and rotation and bias and noise). The evaluation results show that, among a total of seven augmentation cases, four cases including ‘rotation’ (i.e., rotation only, rotation and bias, rotation and noise, and rotation and bias and noise) occupy the top four. Therefore, it may be concluded that the augmentation effect of rotation is overwhelming compared to those of bias and noise. By applying rotation augmentation, the performance of the NN can be significantly improved. The analysis of the effect of the data augmentation techniques presented in this paper may provide insights for developing robust IMU-based orientation estimation networks.

## 1. Introduction

For decades, inertial motion capture has been widely used in various fields to determine the precise three-dimensional (3D) orientation of a moving object without in-the-lab constraints, such as in aerospace [1,2,3], robotics [4], and ambulatory human motion tracking [5,6]. In particular, 3D orientation estimation technology using a nine-axis inertial measurement unit (IMU), which consists of a 3-axis accelerometer, 3-axis gyroscope, and 3-axis magnetometer, is a key technology for inertial motion capture systems.

Conventionally, the nine-axis IMU-based 3D orientation can be estimated through the strap-down integration of the gyroscope signal and the reference directions provided by the accelerometer and magnetometer signals (i.e., the direction of the gravitational acceleration and the direction of the Earth’s local magnetic field, respectively). However, each reference direction can be obtained only in specific conditions (i.e., a static status for the vertical direction and a magnetically homogeneous condition for the horizontal direction). On the other hand, results of the strap-down integration are very vulnerable to the bias of the gyroscope. To this end, a wide variety of sensor fusion algorithms, such as Kalman filters and complementary filters, have been proposed for decades to estimate the 3D orientation by fusing three sensor signals [7,8,9,10].

Recently, with the rapid development of computer hardware, deep learning has been utilized in various applications and has achieved remarkable results. In particular, they are widely used in machine vision and natural language processing to perform image classification, segmentation, text generation, and machine translation tasks [11,12,13,14].

In the last several years, influenced by the successful utilization of deep learning in various applications, methods using deep learning for orientation estimation have been proposed [15,16,17]. Specifically, instead of conventional filter algorithms, alternative approaches have been developed by training neural networks (NNs) end-to-end with a large number of datasets, including raw nine-axis IMU signals and ground truth orientation data [18,19,20,21,22,23]. These studies achieved promising results by showing that NN can estimate orientation with better performance than conventional filters under various conditions.

In deep learning, various factors such as parameter tuning, the architecture of the neural network, and the optimizer affect the performance of the network. Thus, a state-of-the-art training algorithm or network architecture is required to improve the performance. However, the limited amount of training data is a major problem in the development of robust networks. To develop a robust network, particularly for a deep network with a large number of parameters, sufficient training data with distributed characteristics are required. However, in general, collecting large and varied training data is a costly and time consuming process. Therefore, there is a need for an efficient approach to obtaining various training data without performing a data collection process in network training.

Data augmentation, which increases the limited amount of training data based on domain knowledge, is a key approach to addressing the data shortage problem. It is also used to prevent generalizability of the trained network. This approach artificially transforms the original data while maintaining identical characteristics to increase the amount of data. It is commonly used for text or image data, and has been shown to improve network performance [24,25,26,27]. Therefore, the importance of data augmentation in orientation estimation networks has been increasingly emphasized.

To effectively utilize data augmentation in an IMU data-based orientation estimation network, it is important to investigate the effect of augmentation techniques on network performance. Weber et al. [21] used data augmentation techniques to increase the amount of IMU data for orientation estimation, but did not explicitly investigate the effect of data augmentation on the performance of neural network-based orientation estimation. Some studies have applied data augmentation techniques to inertial data and evaluated their effects [28,29,30]. To train a convolutional neural network (CNN) for person identification using inertial data, Tran and Choi [28] introduced data augmentation techniques and investigated their effect on identification performance. Li et al. [29] proposed a data augmentation technique for IMU data to improve the performance of CNN for cattle behavior classification and analyzed its effects. In [30], data augmentation for inertial data was used in long short-term memory (LSTM) for driver’s behavior classification, and the effect of the data augmentation in LSTM was examined. These studies performed data augmentation for recognition and classification by using IMU signals. Data augmentation was applied to inertial sensor data in various fields, and the effect of data augmentation was evaluated. However, to our knowledge, there was no study evaluating the effect of data augmentation on the 3D orientation estimation neural network.

To the best of our knowledge, previous studies on orientation estimation using deep learning focus on developing new and powerful network architectures that can better handle the aforementioned task. That is, studies on the effects of data augmentation techniques on estimation performance in NN-based 3D orientation estimation using IMU signals have not yet been conducted. Thus, the main contribution of this study is that it provides insight into the effect of data augmentation on a 3D orientation estimation network. To this end, a comprehensive experiment was constructed to evaluate the effect of the augmentation technique using a large dataset with distributed conditions. To address the data scarcity problem and improve the robustness, we present three data augmentation techniques (i.e., rotation augmentation, bias augmentation, and noise augmentation) that are widely used in NN-based 3D orientation estimation and analyze the effects of each data augmentation technique on the estimation performance. We trained seven models depending on the augmentation strategy to evaluate their effect on their estimation performance.

The remainder of this paper is organized as follows. In Section 2, we introduce the architecture of the network for 3D orientation estimation, the training algorithm, and three data augmentation techniques applied to the training data. In Section 3, the experimental process for acquiring training and test data, and the training scenario for analyzing the effects of each data augmentation technique are explained. In Section 4, the estimation performance of the trained model using each augmentation technique are compared, and the effects of the augmentation techniques are analyzed.

## 2. Materials and Methods

### 2.1. Three-Dimensional Orientation Estimation Neural Network

To investigate the effects of the data augmentation technique on the orientation estimation network, we first selected the NN architecture. The NN for 3D orientation estimation is a recurrent neural network (RNN) that specializes in time-series data regression and analysis. We utilized the RNN model proposed in [23], which is an extension of the RNN model introduced in [21]. That is, Section 2.1 is a summary of the model proposed in [23].

The RNN architecture is as follows. The input vector of the network is a 9-dimensional vector **x**, which is the 9-axis raw signal of IMU as follows:

(1) x=ySATySGTySMTT

**Figure 1:**
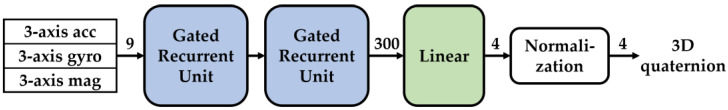
Architecture of the recurrent neural network for 3D orientation estimation.

Here, $y_{A}$, $y_{G}$, and $y_{M}$ are three-dimensional signal vectors of the accelerometer, gyroscope, and magnetometer, respectively. The superscript *S* indicates that the corresponding vector is expressed in the sensor coordinate system, and the superscript T represents the transpose operation. At each sampling time, the input vector is transformed into a 300-dimensional state vector using a two-layer gated recurrent unit (GRU) with 300 neurons per layer. Here, GRU, which is a type of RNN, was developed to deal with the long-term dependency problem of RNN [31]. Finally, for dimensional reduction, the 300-dimensional state vector is transformed into a 4-dimensional vector through a linear layer (also known as a 1-D convolutional layer). To ensure that the output of the network is a unit quaternion representing a 3D orientation, the 4-dimensional vector is normalized inside the network. The overall architecture of the RNN is visualized in Figure 1. 

In the training process, the NN minimizes the error between the estimated quaternion and reference quaternion. At an arbitrary sampling time *t*, the error quaternion $q_{e}(t)$ between the estimated unit quaternion through the network $\hat{q}(t)$ and the reference orientation quaternion qSIref(t), which is the sensor coordinate system *S* with respect to the inertial coordinate system *I*, can be expressed as follows:

(2) qe(t)=qSIref(t)⊗q^(t)−1=[qwqxqyqz]T

Here, ⊗ is a quaternion product, $q_{w}$ is the scalar part of the quaternion, and ${\left[{\begin{array}{ccc} q_{x} & q_{y} & q \end{array}}_{z}\right]}^{T}$ is the vector part of the quaternion. If $q_{e}(t)$ is given at an arbitrary sampling time, the angle between the two quaternions (i.e., the error) is determined by a scalar part of $q_{e}(t)$, as follows [32]:

(3) $$\theta_{e}(t)=2\arccos\left|q_{w}\right|$$

Utilizing the following scalar value $\theta_{e}(t)$ as the error term, the loss function for training was set as the mean squared error (MSE).

The following algorithms were used for network training. If the RNN backpropagates long sequence time-series data (i.e., a large number of samples) to update its weights, it causes vanishing or exploding gradient problems. Therefore, truncated backpropagation through [33] time was used to address this problem. This algorithm splits long-connected sequence data into mini-batches of short sequences and maintains the last hidden state between the mini-batches. The optimization algorithm used for training was Ranger [34], which combines two optimization algorithms: RAdam and Lookahead. We used two training algorithms for tuning the learning rate. One is the one-cycle learning rate policy [35] for fast convergence of the loss function and the other is the learning rate finder [36] for adopting the optimal maximum learning rate. Network implementation and training were performed in the Google Colab environment using fastai v2 API [37] based on PyTorch.

The number of hyperparameters that we set for the training is three. The first one is the sequence length for the truncated backpropagation and was set to 200. The second one is the epoch, which is the number of training iterations and was set to 300. The last one is the batch size, which was set to 64. The learning rate was determined through a learning rate tuning algorithm. 

### 2.2. Data Augmentation Techniques

To improve the estimation performance of the 3D orientation estimation model and its robustness in various environments, we introduced three augmentation techniques that can be applied to IMU-based orientation data. The augmentation technique transforms the original data to generate virtual data with characteristics identical to those of the original data.

The first data augmentation technique used to increase the size of a dataset is rotation augmentation. In this approach, augmentation is performed by virtually rotating the nine-axis IMU signals (i.e., $y_{A}$, $y_{G}$, and $y_{M}$), which are used as input vectors for the network, and the reference orientation qSIref, which is used as the target value. For virtual rotation, we randomly generated a unit quaternion qS′S representing the relative orientation of the virtual sensor coordinate system $S^{\prime }$ with respect to the actual sensor coordinate system. Each 3-axis sensor signal is rotated through a relative orientation quaternion and expressed with respect to the virtual sensor coordinate system *S*′ as follows:

(4) yS′=qS′S−1⊗yS⊗qS′S

Using (4), all three signal vectors expressed in the actual sensor coordinate system are expressed in the virtual sensor coordinate system. Therefore, the input vector generated through rotation augmentation is yS′ATyS′GTyS′MTT. In addition, through virtual rotation, the reference orientation quaternion is rotated to the reference quaternion representing the virtual sensor coordinate system with respect to the inertial coordinate system as follows:

(5) qS′Iref=qSIref⊗qS′S

An arbitrary unit quaternion for the virtual rotation was randomly generated for all experimental trial data. Therefore, each trial datum has a different value. Rotation augmentation is equivalent to tilting the original sensor orientation by an arbitrary constant angle; that is, it has the effect of attaching the sensor to a rigid body in the same location but in a different orientation. Unlike other general augmentation techniques, rotation augmentation transforms the target data as well as the input data. Therefore, when data are transformed through rotational augmentation, virtual data that perform a different type of motion from the original data are obtained.

The second data augmentation technique is bias augmentation, which adds an arbitrary bias value to the gyroscope signal. The basic process of estimating 3D orientation is to perform strap-down integration using the gyroscope signal. However, when the gyroscope signal is integrated, its bias is also integrated, causing boundless orientation drift errors. Therefore, for robust performance against gyroscope bias, data augmentation was performed by adding an arbitrary bias to the gyroscope signal. A randomly generated three-dimensional constant vector **b**, corresponding to the arbitrary bias, is added to the gyroscope signal as follows:

(6) ySG,biased=ySG+b

To apply the virtual gyroscope bias differently to all the experimental data, a constant bias vector was randomly generated for each trial. In bias augmentation, the only difference between the original data and the virtually generated data is the gyroscope signal.

The measurement noise included in each sensor signal is also a factor that increases the estimation error. Therefore, the last augmentation technique is noise augmentation, which adds a virtual measurement noise to the sensor signal. Assuming that the noise of each sensor signal follows white Gaussian noise, virtual Gaussian noise is added to the accelerometer, gyroscope, and magnetometer signals as follows.

(7) $$y_{A,noise}=y_{A}+n_{A}$$

(8) $$y_{G,noise}=y_{G}+n_{G}$$

(9) $$y_{M,noise}=y_{M}+n_{M}$$

Here, $n_{A}$, $n_{G}$, and $n_{M}$ are virtual triaxial Gaussian noises of accelerometer, gyroscope, and magnetometer with zero mean and arbitrary standard deviations, respectively. Because the noise levels (i.e., the standard deviation of noise) of the accelerometer, gyroscope, and magnetometer are different, we generated virtual Gaussian noise according to the noise level of each sensor. Similar to the bias augmentation, the standard deviation of the virtual noise was randomly generated for each trial data.

The following three data augmentation techniques are applied to the training dataset for network training, then we investigate the effect of each augmentation technique on the 3D orientation estimation performance in the RNN.

## 3. Experiment and Training Scenario

### 3.1. Experiment

**Figure 2:**
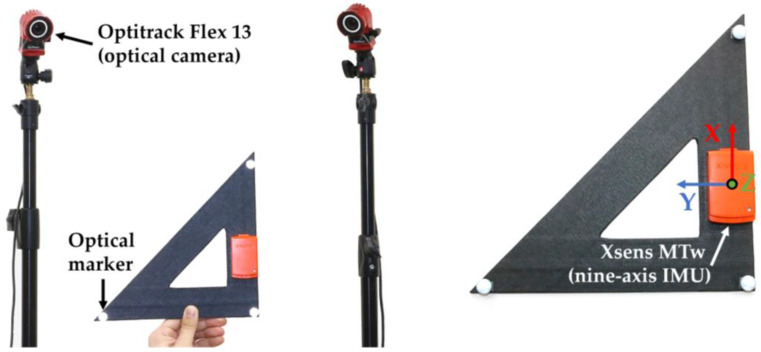
Experimental setup.

An experiment was conducted to acquire a large dataset including the nine-axis IMU signal and reference orientation data. Figure 2 shows the experimental environment. A nine-axis IMU module MTw (Xsens Technologies B. V., Enschede, The Netherlands) consisting of an accelerometer, a gyroscope, a magnetometer, and an optical camera system Optitrack Flex 13 (Natural Point, Corvallis, OR, USA) were used for the experiment. Both systems were sampled at 100 Hz for data acquisition. The IMU sensor was attached to a rigid triangular ruler. The local coordinate system of the rigid body can be created using the three markers attached to each vertex of the rigid body, which was used as the reference orientation of the sensor. However, the relative orientation between the actual coordinate system of the sensor and the local coordinate system of the rigid body obtained using the optical camera may increase the error when evaluating the accuracy of the orientation estimation. To align the two coordinate systems, the quaternion-based local frame alignment method proposed in [38] was used.

The experiment was conducted by randomly shaking the sensor-attached rigid body by hand. All experiments were conducted for approximately three minutes, with static periods of 20 s at the beginning and 10 s at the end. That is, each trial data has approximately 18,000 samples. To train the RNN model through various types of motions, experiments were performed using various criteria to ensure that the dataset included a wide range of motion characteristics. Trials can be divided into two criteria. The first is the criterion for limiting the motion of the sensor, which consists of the following three conditions:

- Rotation: only rotation is performed while maintaining the position of the sensor as much as possible.
- Translation: only translation is performed while maintaining the orientation of the sensor as much as possible.
- Combined: rotation and translation are performed randomly.

The second criterion is the speed of motion. The experiment was conducted according to these criteria by dividing it into fast and slow conditions.

To train and verify the RNN model, a dataset containing a large amount of experimental data was required. Therefore, the 3-minute experiment trial was repeated according to each condition. The dataset comprised 123 trials. The 123 trials were divided into a training dataset for network training, and a test dataset to verify the trained model. A total of 31 trials were used for training, and the remaining 92 were used as test datasets.

### 3.2. Training Scenario

We evaluated the gyroscope bias and noise of the experimental dataset to apply an appropriate level of bias and noise augmentation to the data. The bias and noise were measured in the static state of all trials. The mean of the gyroscope bias magnitudes for all trial data was 0.28 deg/s. For bias augmentation, a constant bias vector **b** was randomly generated from a Gaussian distribution with zero mean and a standard deviation of 0.5 deg/s so that the network has robust performance for larger gyroscope bias. For all trial data, the mean magnitudes of the standard deviation of the accelerometer, gyroscope, and magnetometer noise (i.e., noise level) were 0.02 m/s^2^, 0.15 deg/s, and 0.004 a.u. (arbitrary unit), respectively. The virtual noise is added to each sensor signal according to the noise level of each sensor. Therefore, for each sensor, we randomly generated a three-dimensional constant vector from a Gaussian distribution with zero mean and standard deviation of the noise level of the sensor. Then, each three-dimensional constant vector is used as the standard deviation of the virtual Gaussian noises $n_{A}$, $n_{G}$, and $n_{M}$.

**Figure 3:**
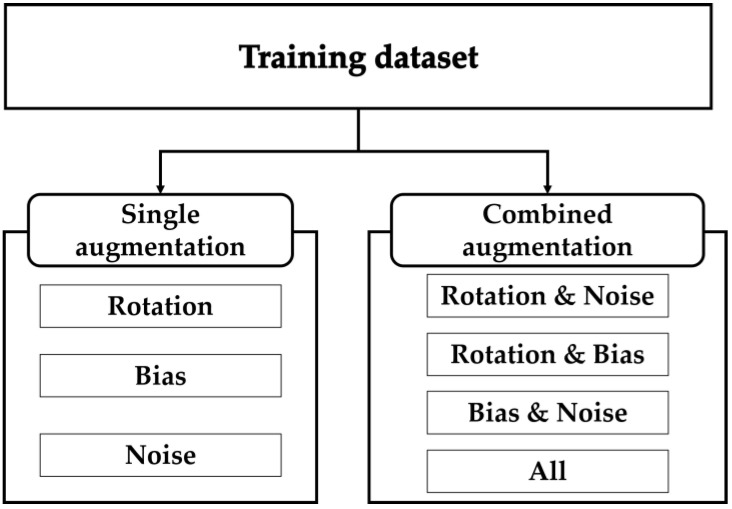
Various data augmentation schemes for training data.

To analyze the effects of the three augmentation techniques on network-based 3D orientation estimation in various aspects, the RNN was trained with a training dataset where the three augmentation techniques were applied individually or in combination. The training dataset, in which data augmentation was applied, included an original dataset and an augmented dataset. Therefore, the size of the training dataset was doubled compared with that of the original dataset. Seven models were trained with the training dataset created using various combinations of the three data augmentation techniques, as follows (see Figure 3):

1. Rotation: An RNN model trained with a training dataset in which only rotation augmentation was applied.
2. Bias: An RNN model trained with a training dataset in which only bias augmentation was applied.
3. Noise: An RNN model trained with a training dataset in which only noise augmentation was applied.
4. Rotation and Bias: An RNN model trained with a training dataset in which both rotation and bias augmentation were simultaneously applied.
5. Rotation and Noise: An RNN model trained with a training dataset in which both rotation and noise augmentation were simultaneously applied.
6. Bias and Noise: An RNN model trained with a training dataset in which bias and noise augmentation were simultaneously applied.
7. All: An RNN model trained with a training dataset in which all three augmentations were simultaneously applied.

**Figure 4:**
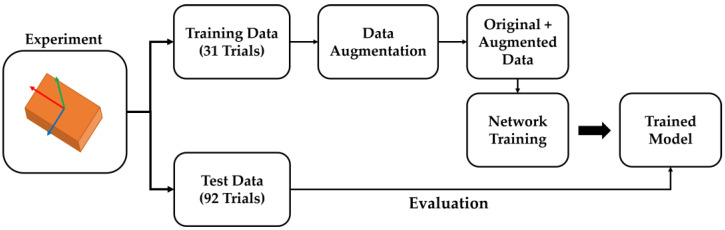
The overall flowchart of network training and evaluation.

In the training process of each model, all the training algorithms were used identically, and only the training dataset was set differently. In addition, the number of epochs, which refers to the number of cycles through the entire training dataset, was set to 300. Figure 4 shows the overall flowchart of the network training and verification for the analysis of the effects of data augmentation. Owing to random factors, such as weight initialization, the performance of the model may be different even when training is performed with the same training parameters. Therefore, the training process of each model was repeated five times, and the mean of the five average root mean square error (RMSE) values over all the test data was used for performance comparison.

## 4. Results and Discussion

To analyze the effects of the data augmentation technique on the orientation estimation performance, each RNN model trained with the seven training datasets was evaluated with a test dataset that was not experienced during the training process. The performance of each model was compared and analyzed using the mean of the RMSEs over all test data. The 3D orientation can be divided into an attitude representing the inclination angle for the gravitational direction and a heading representing an azimuth angle for the direction of the magnetic field of the Earth. Because the two components can be estimated independently, their estimation performance was evaluated independently using the method introduced in [32].

Table 1 shows the orientation estimation performance of the seven network models trained with different augmented training datasets for the test dataset. To quantitatively evaluate the improvement in estimation performance according to each data augmentation technique, the model trained using the augmented training dataset was compared to a model trained using only the original dataset. The estimation performance improvement rate is the average of the performance improvement rates of the attitude and heading angles. The estimation performance of the network model trained with the training dataset containing the augmented data was significantly improved from a minimum of 11.4% to a maximum of 35.2% compared to the RNN model trained using only the original dataset. In addition, the improvement in estimation performance through data augmentation showed an effect on both the attitude and heading angles. That is, all augmentation techniques improved the performance of the orientation-estimation RNN.

We evaluated the effect of each augmentation technique on the estimation performance. When comparing the three models trained on a training dataset with only a single augmentation technique applied, the average improvement rate of the model trained using rotation augmentation was the highest at 30.4%, and the lowest improvement was 11.4%, which was that of the model trained using noise augmentation. In addition, when comparing the three RNN models that simultaneously applied the two augmentation techniques, the improvement in the estimation performance of the two RNN models (28.5% and 27.0%) trained with the rotation augmentation technique was superior to that of the model trained using bias and noise augmentation (21.2%). The improvement rate of the RNN model trained using all three augmentation techniques was 35.2%, showing the best estimation performance among all seven network models. These results indicate that none of the three augmentation techniques adversely affected network training. In addition, these results confirm that training the neural network by applying rotational augmentation has the greatest effect on the improvement of the 3D orientation estimation performance of the network.

One of the reasons for increasing the IMU and reference orientation data through the data augmentation technique is to ensure robust performance against gyroscope bias or sensor measurement noise. Therefore, the three augmentation techniques were individually applied to the test dataset to evaluate the effect of each technique on the three augmentation situations. 

Table 2 shows the estimation performance of each RNN model when three augmentation techniques were applied to the test data: (a) when virtual gyroscope bias was added to the test data, (b) when virtual noise was added to the test data, (c) when the test data were virtually rotated, (d) when unseen data from [39] which are openly available were applied.

In the virtual bias or noise-applied test dataset (see (a) and (b) in Table 2), even if virtual gyroscope bias or measurement noise is added to the original test data, the performance was the same as the estimated result over the original test dataset (see Table 1). That is, the estimation performance of the network did not degrade because of bias or noise. In addition, note that the estimation performance of the model trained with bias or noise augmentation did not improve over the model trained without bias or noise augmentation on the virtual bias or noise-applied test dataset. In the case of mathematical modelling-based filter algorithms, gyroscope bias and measurement noise have a significant effect on performance degradation. However, the above results show that in the case of NNs, even if the network is trained with only the gyroscope bias and noise included in the original data, it can sufficiently maintain a robust performance against larger bias and noise.

In the virtually rotated test dataset (see (c) in Table 2), the estimation performance of all models was significantly degraded. In particular, the original model showed the highest error of more than 30° for both the attitude and heading. In addition, the models trained without rotation augmentation showed performance at a level where orientation estimation was impossible, with an attitude error of more than 25° and a heading error of more than 31°. Similarly, for the models trained with rotation augmentation, the estimation performance was significantly degraded with an estimation error of more than 10°. The reasons for such significant degradation of the estimation performance on virtually rotated data are as follows. To obtain the training and test datasets, all experiments were conducted including the static state for the initial 20 s and the last 10 s. For the static state, the sensor was placed on a table with the *z*-axis pointing upward so that the sensor could maintain a static state. The network model, which was trained without rotation augmentation data, was trained using biased training data in which the *z*-axis of the sensor pointed upward while the sensor was in a static state. However, in the case of virtually rotated test data, the sensor is placed in an arbitrary orientation even in a static state. Thus, when estimating the orientation of the sensor, the estimation performance of the model trained with biased experimental data is significantly reduced. In other words, it causes an overfitting problem during the training process. In addition, models trained with a dataset in which rotation augmentation was applied similarly showed poor estimation performance, as shown in the above results, because half of the training dataset was original data. 

With regard to the results shown in (d) in Table 2, an openly available dataset from [39] was used in order to examine the tendency of the effect of data augmentation on unseen data. In terms of estimation accuracy, the case for the unseen dataset produced worse results than the other cases shown in (a)–(c) in Table 2. However, since this paper deals not with estimation performance but instead with the effects of data augmentation on the estimation performance, an in-depth discussion of estimation performance itself is out of the scope of this paper.

Most importantly, the evaluation results in Table 1 and Table 2 show that, among a total of seven augmentation cases (see Figure 3), four cases including ‘rotation’ (i.e., rotation only, rotation and bias, rotation and noise, and rotation and bias and noise) occupy the top four. Therefore, it may be concluded that the augmentation effect of rotation is overwhelming compared to those of bias and noise. Furthermore, it can be observed that, among the four cases including ‘rotation’, the case of applying ‘rotation and bias and noise’ shows superior performance over the other three cases. This indicates that, no matter how overwhelming the effect of rotation is, augmentation by adding bias and noise is (even a little) better than augmentation only by rotation.

**Figure 5:**
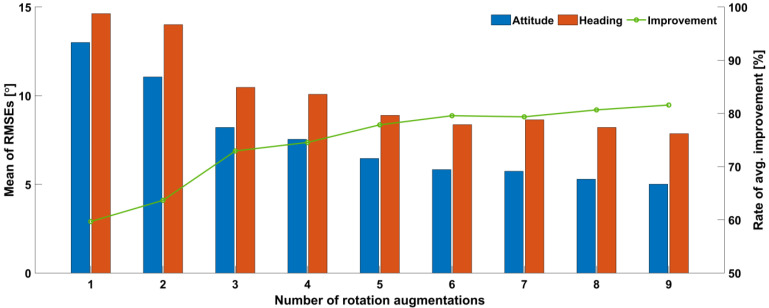
Estimation performance (left: mean of RMSEs for attitude and heading, right: rate of averaged improvement) of virtually rotated test data according to the number of rotation augmentations.

To specifically analyze the effect of rotation augmentation, we trained the RNN network by gradually increasing the size of the training dataset, applying rotation augmentation to the training data many times and evaluating the model performance according to the number of augmentations. Therefore, rotation augmentation was applied to the training dataset incrementally up to nine times. The size of the training dataset with rotation augmentation applied nine times was ten times larger than the size of the original training dataset. Each trained model was evaluated over the same virtually rotated test data as listed in (c) in Table 2. Figure 5 shows the estimation performance according to the number of rotation augmentations applied to the training dataset and the performance improvement rate compared to the model trained with the original dataset. As the number of applications of rotation augmentation increased, the estimation performance further improved. When rotation augmentation is applied once, the improvement rate increased the most (59.67%). As the application number of augmentation increased, the performance improvement rate decreased, and from the seventh application, the improvement rate decreased to less than 1%. These results indicate that there is a limitation in the performance improvement through data augmentation in the orientation estimation RNN. The average RMSE of the model trained applying rotation augmentation nine times was 5.01° and 7.86° for attitude and heading, respectively. This model outperformed all other models (see Table 1), which was evaluated with the original test data. The following results indicate that it is very important to properly use the rotation augmentation technique for the estimation performance of the 3D orientation estimation RNN.

## 5. Conclusions

This study analyzed the effects of three data augmentation techniques on nine-axis IMU-based 3D orientation estimation performance in an RNN. The three augmentation techniques are rotation augmentation, which virtually rotates the IMU signal and reference orientation; bias augmentation, which adds an arbitrary gyroscope bias; and noise augmentation, which adds virtual measurement noise to each sensor. To investigate the effect of each augmentation technique on estimation performance, seven training datasets were created by combining the three augmentation techniques, and the RNN model proposed in [23] was trained with each training dataset. The validation results showed that among the three augmentation techniques, rotation augmentation had the greatest effect on improving the estimation performance of orientation RNN. In addition, by applying rotation augmentation, the performance of the neural network can be significantly improved.

As a main contribution of this study, we quantitatively investigated the improvement in the estimation accuracy of network-based 3D orientation using data augmentation. To the best of our knowledge, studies on the effects of data augmentation techniques on estimation performance in orientation estimation networks using IMU sensors have not yet been conducted. In this regard, the analysis of the effect of the data augmentation techniques presented in this paper can provide insights for developing robust IMU-based orientation estimation networks. In future works, we aim to develop an IMU-based human motion tracking system based on a NN-based orientation estimation, and investigate the performance difference between conventional filter-based estimation and NN-based estimation in terms of motion tracking accuracy.

## Figures and Tables

**Table 1:** Estimation performance (mean of RMSEs) for each RNN model over all the test data (unit: °).

	Attitude	Heading	Avg. Improvement
Original	9.27	12.11	
Rotation	6.59	8.26	30.4%
Bias	7.32	9.49	21.3%
Noise	7.98	11.03	11.4%
Rotation and Bias	6.63	8.65	28.5%
Rotation and Noise	6.60	9.05	27.0%
Bias and Noise	7.35	9.47	21.2%
All	5.99	7.86	35.2%

**Table 2:** Estimation performance (mean of RMSEs) for each RNN model over three augmented test datasets (unit: °).

(**a**) Results for the test dataset with applied virtual gyroscope bias			
	**Attitude**	**Heading**	**Avg. Improvement**
Original	9.27	12.09	-
Rotation	6.60	8.25	30.3%
Bias	7.33	9.47	21.3%
Noise	7.98	11.02	11.4%
Rotation and Bias	6.63	8.64	28.5%
Rotation and Noise	6.60	9.04	27.0%
Bias and Noise	7.37	9.45	21.2%
All	6.00	7.85	35.2%
(**b**) Results for the test dataset with applied virtual noise			
	**Attitude**	**Heading**	**Avg. Improvement**
Original	9.27	12.11	-
Rotation	6.59	8.26	30.4%
Bias	7.32	9.49	21.3%
Noise	7.98	11.03	11.4%
Rotation and Bias	6.63	8.65	28.5%
Rotation and Noise	6.60	9.05	27.0%
Bias and Noise	7.36	9.47	21.2%
All	5.99	7.85	35.2%
(**c**) Results for the test dataset with applied virtual rotation			
	**Attitude**	**Heading**	**Avg. Improvement**
Original	30.43	38.57	-
Rotation	13.00	14.63	59.7%
Bias	25.98	33.79	13.5%
Noise	27.24	35.99	8.6%
Rotation and Bias	12.36	15.77	59.3%
Rotation and Noise	11.58	14.31	62.3%
Bias and Noise	25.81	31.95	16.2%
All	11.60	14.45	62.2%
(**d**) Results for the dataset from [39]			
	**Attitude**	**Heading**	**Avg. Improvement**
Original	31.49	54.77	-
Rotation	26.40	31.24	29.6%
Bias	30.90	45.16	9.71%
Noise	33.72	50.82	0.05%
Rotation and Bias	24.77	32.69	30.8%
Rotation and Noise	23.74	34.71	30.6%
Bias and Noise	34.36	49.01	0.69%
All	24.63	31.28	32.3%

## Data Availability

The data presented in this study are available on request from the corresponding author.
